# The Mutational Landscape of SARS-CoV-2

**DOI:** 10.3390/ijms24109072

**Published:** 2023-05-22

**Authors:** Bryan Saldivar-Espinoza, Pol Garcia-Segura, Nil Novau-Ferré, Guillem Macip, Ruben Martínez, Pere Puigbò, Adrià Cereto-Massagué, Gerard Pujadas, Santiago Garcia-Vallve

**Affiliations:** 1 Departament de Bioquímica i Biotecnologia, Research Group in Cheminformatics & Nutrition, Campus de Sescelades, Universitat Rovira i Virgili, 43007 Tarragona, Spain; bsaldivar.emc2@gmail.com (B.S.-E.); polgarse2@gmail.com (P.G.-S.); nnovauf@gmail.com (N.N.-F.); guillem.macip@gmail.com (G.M.); 2 Institut La Guineueta, 08042 Barcelona, Spain; rmartbernabe@gmail.com; 3 Department of Biology, University of Turku, 20500 Turku, Finland; pepuav@utu.fi; 4 Department of Biochemistry and Biotechnology, Rovira i Virgili University, 43007 Tarragona, Spain; 5 Eurecat, Technology Centre of Catalonia, Unit of Nutrition and Health, 43204 Reus, Spain; 6 EURECAT Centre Tecnològic de Catalunya, Centre for Omic Sciences (COS), Joint Unit Universitat Rovira i Virgili-EURECAT, Unique Scientific and Technical Infrastructures (ICTS), 43204 Reus, Spain; ssorgatem@gmail.com

**Keywords:** SARS-CoV-2 mutations, COVID-19, molecular evolution

## Abstract

Mutation research is crucial for detecting and treating SARS-CoV-2 and developing vaccines. Using over 5,300,000 sequences from SARS-CoV-2 genomes and custom Python programs, we analyzed the mutational landscape of SARS-CoV-2. Although almost every nucleotide in the SARS-CoV-2 genome has mutated at some time, the substantial differences in the frequency and regularity of mutations warrant further examination. C>U mutations are the most common. They are found in the largest number of variants, pangolin lineages, and countries, which indicates that they are a driving force behind the evolution of SARS-CoV-2. Not all SARS-CoV-2 genes have mutated in the same way. Fewer non-synonymous single nucleotide variations are found in genes that encode proteins with a critical role in virus replication than in genes with ancillary roles. Some genes, such as spike (*S*) and nucleocapsid (*N*), show more non-synonymous mutations than others. Although the prevalence of mutations in the target regions of COVID-19 diagnostic RT-qPCR tests is generally low, in some cases, such as for some primers that bind to the *N* gene, it is significant. Therefore, ongoing monitoring of SARS-CoV-2 mutations is crucial. The SARS-CoV-2 Mutation Portal provides access to a database of SARS-CoV-2 mutations.

## 1. Introduction

Mutation (including insertions and deletions) and recombination are two important mechanisms that generate genomic variability in SARS-CoV-2 variants [1]. Most SARS-CoV-2 mutations are expected to be either neutral or mildly deleterious [2]. Highly deleterious mutations, such as those that prevent the virus from invading the host, are unlikely to occur. However, SARS-CoV-2 is under selective pressure because of vaccines and antiviral drugs [3]. Mutations that improve virulence, infectivity, transmissibility, increase viral replication, or aid in immune evasion are expected to be fixed and spread. However, the high frequency of certain mutations is not always due to a mutation’s beneficial effect. It can also be caused by a founder effect, which occurs when a mutation appears early in the evolution of a pandemic and is transmitted to all of its descendants [4] or when a mutation is found in a variant that also carries an additional advantageous mutation. Genetic diversification of the SARS-CoV-2 virus has led to the emergence of new clades and variants [5,6]. Variants of concern (VOC) are SARS-CoV-2 variants for which there is evidence of an increase in transmissibility or virulence, a detrimental charge in COVID-19 epidemiology, or a decrease in the effectiveness of available diagnostic tools, vaccines, or therapeutics. Omicron is the only currently circulating VOC (in March 2023). It was first identified in November 2021 and has since been responsible for the vast majority of COVID-19 cases worldwide [7]. This variant has undergone significant mutations in comparison to previous variants [8]. Alpha, beta, delta, and gamma VOCs have all previously been in circulation. In December 2020, a rapidly growing lineage (the alpha variant) was identified in the UK [1] and increased in prevalence worldwide in the following months. Soon after, other rapidly growing variants, beta and gamma, appeared [1] but were soon overtaken by the delta variant that appeared in India, spread widely in numerous countries, and become the predominant variant in the second part of 2021 until the emergence of Omicron. All of these variants contained the spike mutation D614G that resulted in increased SARS-CoV-2 infectivity [9,10,11].

Mutations in SARS-CoV-2 can be caused by RNA-dependent RNA polymerase (RdRp) replication errors or by host deaminases that deaminate unpaired nitrogenous bases [1]. At the start of the pandemic, the prevalence of C>U mutations and other evidence suggested that RNA editing is the major source of SARS-CoV-2 mutation [12,13,14,15,16,17]. Since then, the role of RNA editing in SARS-CoV-2 evolution has been experimentally demonstrated [18,19,20]. Because of the prevalence of RNA editing in SARS-CoV-2 evolution, some recurrent mutations in SARS-CoV-2 can be predicted [21]. Mammalian apolipoprotein B mRNA editing catalytic polypeptide-like (APOBEC) enzymes deaminate cytosines into uracils in single-stranded DNA (ssDNA) and ssRNA [22]. When APOBEC enzymes act on the SARS-CoV-2 genome’s negative strand, G>A mutations occur on the positive strand [23]. Adenosine deaminases acting on RNA (ADAR) deaminate adenines into inosines (A>I) in double-stranded RNA (dsRNA) [24]. As inosine preferentially pairs with cytidine, A>I mutations cause A>G and U>C transitions on the positive strand of the SARS-CoV-2 genome [23,25]. The presence of SARS-CoV-2 mutations may affect the test performance of COVID-19 diagnostic tests [26,27,28]. For this reason, they must be monitored. To provide guidelines and recommendations for assessing the potential effects of current and future viral mutations of SARS-CoV-2 on COVID-19 tests, in February 2021 the FDA published the “Policy for Evaluating Impact of Viral Mutations on COVID-19 Tests”, which was updated in January 2023. Molecular tests are intended to detect viruses by focusing on a specific region of the viral genome. False-negative results can occur if there are mutations that reduce the ability of these tests to detect the virus’s RNA genome. The gold-standard test to detect COVID-19 is the quantitative RT-PCR (RT-qPCR), which uses forward and reverse primers to amplify a specific region of the SARS-CoV-2 genome. Probes bind downstream of one of the primers and give a fluorescent signal proportional to the number of amplicons synthesized. Various primers and probe sets have been reported for the detection of SARS-CoV-2 by RT-qPCR [29]. SNVs, mutations, and insertions can affect primer and probe hybridization and cause amplification failure [26], especially if they cause mismatches with the template DNA near the 3’-end of a primer [30]. The effects of mutations are less pronounced in tests designed to detect multiple SARS-CoV-2 genes than in tests designed to detect a single target. For example, the use of a multiplex RT-qPCR made it possible to identify the alpha variant for the first time in the UK. The 69-70 deletion in this variant prevents the spike (*S*) gene from being amplified in the Thermo Fisher TaqPath COVID-19 PCR assay, resulting in *S*-gene target failure in the test results [31].

The study of SARS-CoV-2 mutations is critical for detecting and treating SARS-CoV-2 and developing vaccines, and it must be carried out on a regular basis. Throughout the pandemic, the SARS-CoV-2 mutational landscape has been analyzed recurrently [32,33,34,35,36], though occasionally with a small number of genomes or focusing on amino acid changes or a specific gene, country, or variant. In this article, we analyze the mutational landscape of SARS-CoV-2 using data from more than 5 million SARS-CoV-2 genomes, collected after more than two years of the pandemic. We focus on nucleotide-level changes, and in particular, we analyze their distribution among SARS-CoV-2 genes, the most common mutations and types of mutations, and their potential impact on COVID diagnostic tests.

## 2. Results and Discussion

### 2.1. SARS-CoV-2 Genomes Analyzed

**Figure 1:**
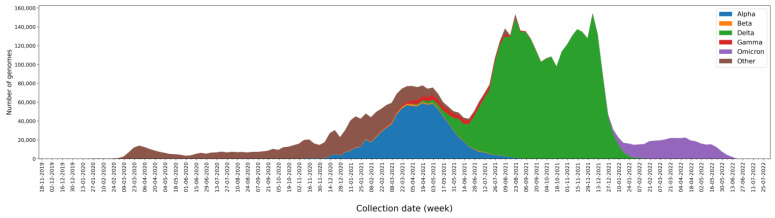
The number of genomes collected per week and classified by a variant of concern (VOC): alpha in blue, beta in orange, delta in green, gamma in red, omicron in purple, and others in brown.

We analyzed 5,340,569 SARS-CoV-2 genomes available from the GISAID database [37]. They are complete, high-coverage SARS-CoV-2 genomes isolated from humans and were available on 27 June 2022. Since the rates of genome sequencing in different nations fluctuate significantly, it is important to keep in mind that there is a bias in the genomes examined. The USA and the United Kingdom sequenced 51.9% of all genomes (Figure S1). In terms of continents, Europe (55.1%) and North America (34.1%) accounted for the majority of genomes (Table S1). However, this bias does not invalidate the results reported herein. The genomes analyzed were collected between December 2019 and June 2022 (Figure 1). The number of genomes increased from 2020 as sequencing efforts in different countries and the number of cases increased. At the end of 2020, the alpha variant emerged, and throughout the first few months of 2021, it predominated, although it did not completely replace earlier varieties. The delta variant caused an exponential rise in the number of cases, and by the end of 2021, it was the most common variety. Then, at the start of 2022, the omicron variant took its place (Figure 1).

### 2.2. Mutations, Deletions, and Insertions per Genome per Week

**Figure 2:**
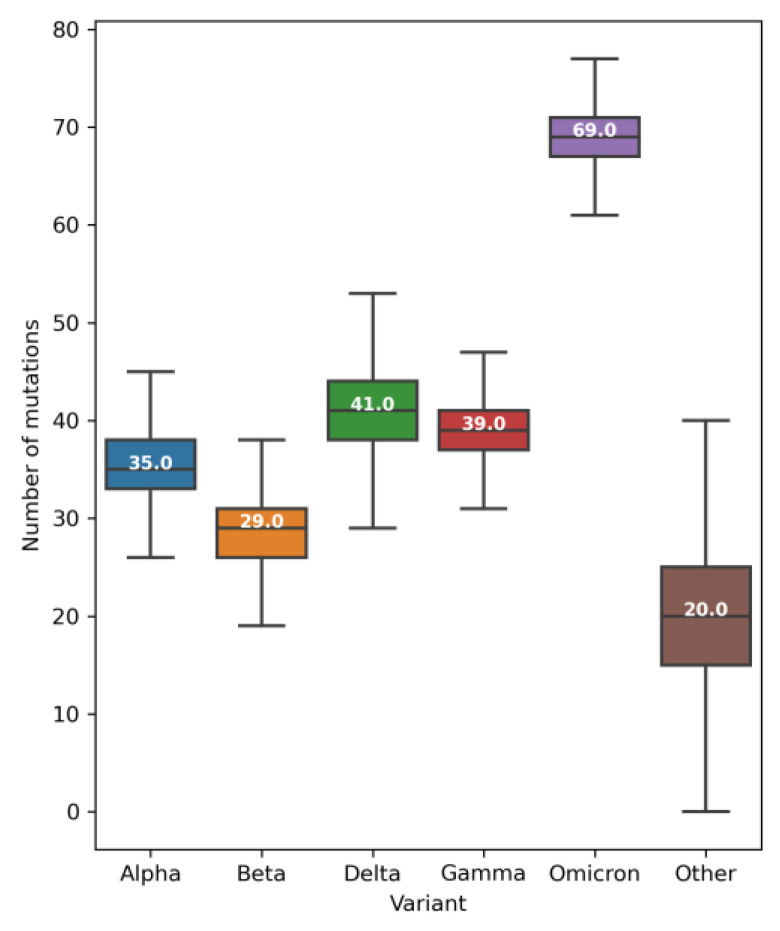
Boxplots of the number of single nucleotide variants (SNVs) per genome and VOC. The boxes show the first, second (median), and third quartiles, and the whiskers show the minimum and maximum values, excluding outliers. The median of each VOC is shown in white.

Among the mutations, the most frequent were single nucleotide variants (SNVs): i.e., those that exchange one nucleotide for another (Table S2). As expected, the number of SNVs per genome per week increased during the pandemic (Figure S2). Until mid-May 2020, the average number of SNVs per genome was less than 10 (Figure S2). In June 2020, the average was around 7 [33] but by the beginning of January 2022, it had increased to 50. It then increased again when the omicron variant expanded, and by early June 2022, the average number of SNVs per genome was around 72 (Figure S2). In terms of variants, alpha, beta, delta, and gamma VOCs contain a median of 29 to 41 SNVs per genome (Figure 2). The omicron variant is the most highly mutated VOC, with over 60 SNVs per genome (Figure 2) that potentially improve transmissibility, immunological evasion, and virulence [38,39].

The number of deletions per genome per week was quite low until early 2021 when there was an increase (Figure S3). Since then, they have remained at an average of three deletions per genome. Some deletions are conserved in SARS-CoV-2 variants and have a significant regional preference, possibly to prevent neutralizing antibodies from binding to their target and thus cause immune escape [40,41,42]. Thus, although SNVs outnumber deletions, deletions have a significant influence on the evolution of viruses and may contribute to the evasion of immune responses and the evolution of highly transmissible variants [43,44]. Over the course of the pandemic, there have been few insertions, an average of 0.2 per genome (Figure S4). Questions have been raised about whether some of the insertions observed in the SARS-CoV-2 genomes were insertions or sequencing artifacts [45]. Figures S5 and S6 show that the most common lengths of deletions and insertions in the coding regions of the SARS-CoV-2 genome are multiples of three nucleotides (3, 6, 9, …). This suggests that some of the deletions and insertions are caused by real viral variation and not by sequencing errors. Single nucleotide deletions are relatively frequent (Figure S5), but 26% of them occur in *ORF7a* or *ORF8* genes. Deletions that truncate the *ORF7a* or *ORF8* genes have been observed and associated with a milder infection [43,46]. Because insertions and deletions can affect the antigenic properties of SARS-CoV-2 proteins, they had to be monitored [40,45].

### 2.3. Most Frequent SNVs

A total of 73,464 different SNVs were found in the 5,340,569 SARS-CoV-2 genomes analyzed. Of these, 1842 were mutations from untranslated regions (UTRs), 51,467 were non-synonymous, 18,413 were synonymous, and 1742 were only observed in conjunction with another mutation affecting the same codon (Table 1). Although there are more non-synonymous than synonymous mutations, synonymous mutations are generally more frequent (Figure S7 and median values in Table 1). This is to be expected because synonymous mutations have fewer restrictions and do not alter the coded protein. However, codon usage and the maintenance of the RNA secondary structure are two forces that can cause some selection pressure on synonymous mutations [47]. The distribution of synonymous mutations and mutations from UTRs are comparable (Figure S7).

**Figure 3:**
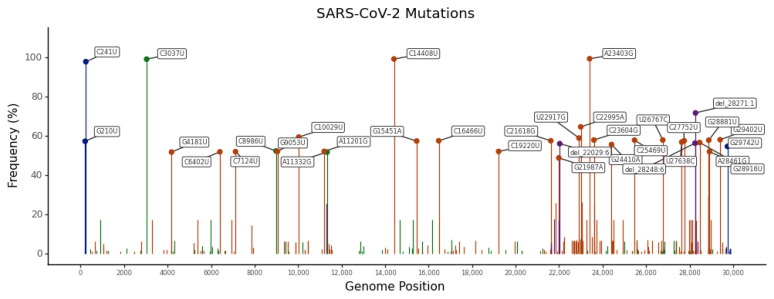
Lolliplot of the most frequent SARS-CoV-2 mutations. Synonymous, non-synonymous, and UTR mutations are shown in green, dark orange, and blue, respectively. Deletions are in purple.

Not all SNVs are equally frequent and many are low frequency [48]. In fact, 23.69%, 8.19%, and 4.61% of SNVs have been found in only one, two, or three genomes (Figure S8). These percentages decrease as the number of genomes increases, but 27.25% of SNVs have been found in more than 100 genomes (Figure S8). The most frequent SNVs are the A23403G spike mutation (present in 99.47% of SARS-CoV-2 genomes analyzed), the C14408U RdRp mutation (present in 99.35% of genomes), the C3037U synonymous mutation (present in 99.27% of genomes), and the C241U UTR mutation (present in 97.96% of genomes) (Figure 3 and Table 2). These mutations appeared early in the pandemic (January 2020) and have since become dominant [33,49]. The A23403G mutation causes the D614G mutation at the S protein, which has been associated with enhanced infectivity [9,10,11]. The C14408U mutation in the *RdRp* gene causes the non-synonymous mutation P323L (Table 2). According to recent studies, this mutation confers transmission advantages and was crucial to the P323L/D614G genotype becoming established early in the pandemic [50]. C3037U and C241U mutations are most likely neutral [51]. C3037U is a synonymous mutation that affects the *nsp3* gene and C241U is found in an unpaired six-base loop in the conserved 5′-UTR SL5B secondary structure [51]. The most frequent mutations in each SARS-CoV-2 gene are shown in Figure S9. Of the 24 genes, 12 (i.e., the *S*, *RdRp*, *nsp3*, *nsp4*, nucleocapsid (*N)*, *M*, *ORF3a*, *helicase*, *ORF7a*, *ORF8*, *nsp6*, and *exonuclease*) have some mutations with a prevalence higher than 50% (Figure S9).

Some of the SARS-CoV-2 mutations are specific to some SARS-CoV-2 variants and have been used for early identification of SARS-CoV-2 variants through amplification [52,53]. Table 3 shows some of the variant-specific mutations from the spike protein and their frequency among some variants. L452R, W152C, K417T, and K417N mutations are particularly specific to Delta, Epsilon, Gamma, and Omicron variants, respectively (Table 3). These and other mutations (and combinations of them) have been proposed to identify variants, but erroneous identifications can occur when using only single specific mutations [52]. Therefore, sequencing is currently the gold standard method for variant identification [52].

**Figure 4:**
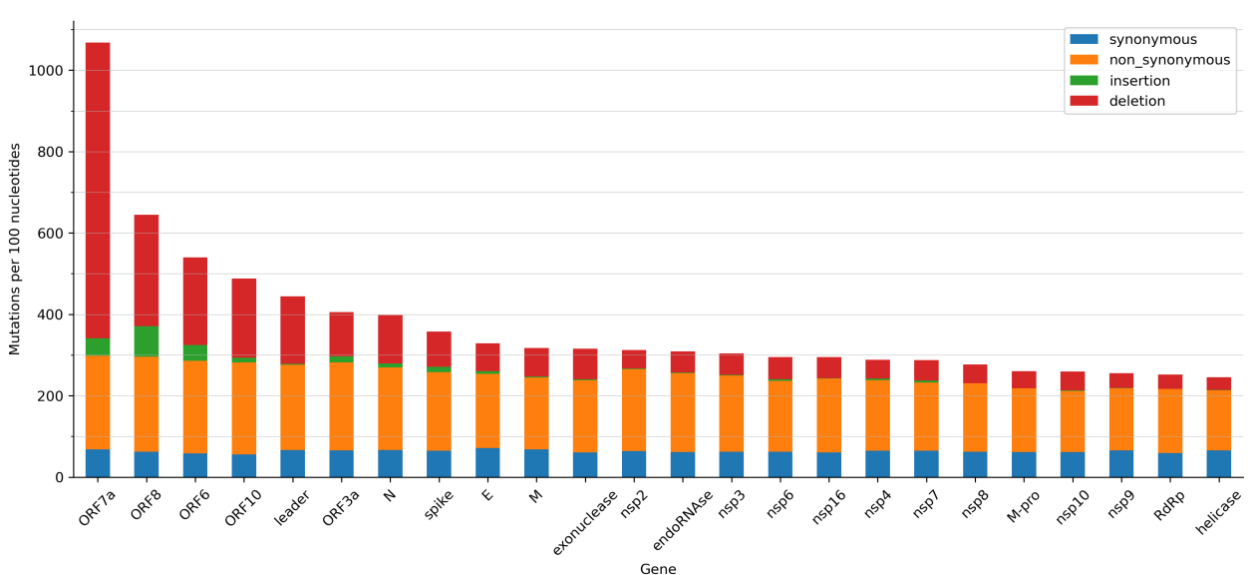
Mutations per 100 nucleotides in the SARS-CoV-2 genes. Synonymous, non-synonymous mutations, insertions, and deletions are shown in blue, orange, green, and red, respectively.

Not all SARS-CoV-2 genes have accumulated the same number of mutations. As mutation rates per nucleotide are small, our calculations were based on 100 nucleotides (Figure 4). The number of synonymous mutations per 100 nucleotides is quite similar across all SARS-CoV-2 genes (Figure 4). On average, there are 63.6 synonymous SNVs per 100 nucleotides. Other types of mutations are more variable. The number of non-synonymous SNVs per 100 nucleotides ranges between 147.5 and 298.5 (Figure 4). There are fewer non-synonymous SNVs in genes that encode proteins that play critical roles in virus replication, e.g., *helicase*, *RdRp*, and main protease (*M-pro*), than in genes with accessory functions (e.g., *ORF7a*, *ORF8*, and *ORF6*). This is consistent with previous observations from mid-2020, which indicates that there is a tendency to conserve important structural and functional features in SARS-CoV-2 proteins [35]. Genes encoding S and N proteins have more non-synonymous SNVs than other genes (Figure 4). We expected the *S* gene to contain more non-synonymous mutations. Mutations in the S protein may enhance its interaction with ACE2, help it to escape from the immune system, or improve furin cleavage [2,3,54,55]. It has also been suggested that the *S* gene is more likely to be single-stranded than other SARS-CoV-2 genes, thus making it a favourable target for C>U deamination and leading to an excessively high mutation rate [56]. The high mutation frequency of the *N* gene may be due to its higher G+C percentage [57]. This gene is frequently used as a target for RT-qPCR diagnostic tests and it has been suggested that it be part of future vaccines against COVID-19 [58]. Nonetheless, its high mutation frequency must be considered since any changes in this gene may render vaccines or diagnostic tests ineffective [59]. However, mutations in the *N* gene are not uniformly distributed, and a leucine-rich sequence (LRS) from amino acids 218 to 231 is a conserved region that may provide a new path for the development of pan-coronavirus therapeutics and vaccines [60,61].

The number of insertions and deletions among SARS-CoV-2 genes is also highly variable (Figure 4). Genes that encode proteins essential for viral replication contain fewer insertions and deletions (Figure 4). It is worth noting a large number of deletions in accessory genes, such as *ORF7a*, *ORF8*, and *ORF6* (Figure 4). It has been suggested that deletions in these genes may eventually lead to more effective variants that produce a milder infection [43,44,46]. In all genes, insertions are less common than deletions (Figure 4).

### 2.4. SNV Signature Analysis

**Figure 5:**
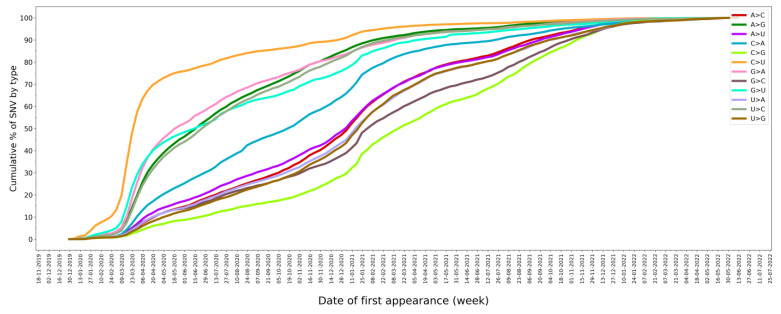
Cumulative percentage of SNV types by date of first appearance.

Of the 73,464 SNVs analyzed, transversions—i.e., an SNV in which a purine is exchanged for a pyrimidine or vice versa—are more frequent than transitions (61.72% vs. 38.28%). The most prevalent mutations are U>C and A>G (Table 4). However, because the SARS-CoV-2 genome is richer in As and Us than in Gs and Cs (its G+C content is 37.97%), the C>U mutation stands out when the fraction of each type of nucleotide that has mutated is calculated (Table 4). A total of 97.4% of all Cs in the SARS-CoV-2 genome have mutated at some time to a U, but only 65.2% of them have mutated to a G (Table 4). This is consistent with the C>U mutation being the most common SNV at the beginning of the pandemic (Figure 5) [15,33,34,62]. By mid-April 2020, 70% of all C>U mutations had already been observed (Figure 5). In addition, C>U mutations are the most frequent mutations on average [17], and they have been observed in the largest number of variants, pangolin lineages, and countries (Figure S10). All of this evidence supports the role of C>U mutations as a driving mechanism in the evolution of SARS-CoV-2 [63]. The second most remarkable SNV type is the A>G mutation (Table 4). A total of 94.0% of all As in the SARS-CoV-2 genome have mutated at some time to a G (Table 4), and 70% of total A>G mutations were first observed by the end of September 2020 (Figure 5). The prevalence of C>U and A>G mutations is consistent with the predominant role of host deaminases in causing a significant portion of SARS-CoV-2 mutations [14,17,18,64]. 

### 2.5. Mutations in the Target Regions of the COVID-19 Diagnostic RT-qPCR Tests

Table 5 and Table S3 show the number of different mutations found in the primer and probe regions used in the RT-qPCR for COVID-19 diagnosis. Although the frequency of mutations is usually low (Figure S11), in some cases they are important. For example, the total frequency of the Charite-RdRp primer/probe set is 60.84% (Table 5), or 57.57% when the SNVs were in the last 5 nucleotides of the 3’-end of the forward primer (Table S3). For the China-CDC-N set, the total frequency is 141.29% (Table 5), mainly due to three missense mutations: (i) the G28881U mutation that is found in 57.8% of the genomes analyzed; (ii) the two simultaneous mutations G28881A and G28882A that affect the same codon, with a frequency of 29.3% and (iii) the G28883C mutation, with a frequency of 28.1%. The *N* gene is highly conserved in coronavirus. For this reason, it has been extensively used by RT-qPCR as a target region to detect COVID-19. However, the *N* gene is one of the SARS-CoV-2 genes with the most reported mutations (Figure 4). Some *N* gene mutations, such as the SNVs G29140U, G29179U, and C29200U, and deletions have been reported to affect RT-qPCR results [65,66,67,68,69,70,71,72]. Therefore, using primers and probes that hybridize to a region of the *N* gene is not an optimal choice [73]. A negative result in one of the target genes in a multiplex RT-qPCR assay used to detect COVID-19 is not interpreted as a negative test result, but it may render the assay susceptible to diagnostic failure. Consequently, continued surveillance of SARS-CoV-2 mutations is critical [74]. However, the lack of information about the primers and probes used by some commercial RT-qPCR kits is a drawback for this type of analysis. To reduce the impact of SARS-CoV-2 mutations on COVID-19 surveillance, new primers, and probes targeting the most conserved regions of the SARS-CoV-2 genome or specific regions of a SARS-CoV-2 variant have been suggested [74].

### 2.6. SARS-CoV-2 Mutation Portal

We have created a database of all the mutations discovered in the more than five million SARS-CoV-2 genomes analyzed. The SARS-CoV-2 Mutation Portal (http://sarscov2-mutation-portal.urv.cat/, accessed on May 2023) provides access to this database, which contains information on over 100,000 mutations (including point mutations, insertions, and deletions). For each mutation, it gives a variety of information, such as the type of mutation, its location, effect, frequency, the number of countries, lineages, and variants in which it has been found. The mutations are shown in the form of a table and a scatter diagram (Figures S12–S14).

## 3. Materials and Methods

### Origin and Characterization of the SARS-CoV-2 Genomes Analyzed

A FASTA file containing the multiple sequence alignment of 10,417,619 complete SARS-CoV-2 genomes were downloaded from the GISAID database [37] on 27 June 2022. In this multi-alignment file, the SARS-CoV-2 sequence NC_045512.2, isolated from Wuhan and submitted to the GenBank database on 17 January 2020, was used as a reference. Only sequences labelled as “high coverage” (i.e., sequences containing: (a) less than 1% of unidentified bases (Ns), (b) less than 0.05% of unique amino acid mutations, to withdraw possible sequencing artefacts, and (c) no insertions and/or deletions, unless verified by the submitter) and obtained from human samples were considered. Thus, the initial number of SARS-CoV-2 genomes was reduced to 5,340,569 sequences. For each sequence, information about the collection date, location, pango lineage [75], and VOC was extracted from a metadata file available in GISAID. For each sequence, single mutations, insertions, and deletions were extracted and numbered relative to the reference genome. Mutations were classified as mutations from UTRs, synonymous mutations (i.e., mutations that do not affect the encoded amino acid), and non-synonymous mutations (which include missense and nonsense mutations). Mutation frequencies were calculated as the number of specific mutations in the total number of genomes. All analyses and figures were created with custom programs in Python 3.9.

## 4. Conclusions

Although almost every nucleotide in the SARS-CoV-2 genome has mutated at some time, the frequency and regularity of the mutations vary significantly. C>U mutations are the most prevalent mutations. They are found in the largest number of variants, pangolin lineages, and countries. The predominance of C>U mutations during the early stages of the pandemic suggested that host deaminases were responsible for a considerable percentage of SARS-CoV-2 mutations. Since then, the predominant role of host deaminases on SARS-CoV-2 evolution has been demonstrated experimentally. Not all SARS-CoV-2 genes have accumulated the same number of mutations. Non-synonymous SNVs are less common in genes encoding proteins that have key roles in virus replication than in genes with accessory functions. Genes encoding S and N proteins are among the genes with the most non-synonymous SNVs. Although the prevalence of mutations in the target regions of COVID-19 diagnostic RT-qPCR tests is generally low, it is significant in some cases, such as for some primers that bind to the *N* gene. For this reason, SARS-CoV-2 mutations must be tracked. However, the lack of information about the primers and probes used by some commercial RT-qPCR kits is a drawback for this type of analysis. The SARS-CoV-2 Mutation Portal (at http://sarscov2-mutation-portal.urv.cat/, accessed on 10 May 2023) gives access to a database of all the mutations (including point mutations, insertions, and deletions) that have been analyzed here. 

## Figures and Tables

**Table 1:** Unique SNV counts and the median number of genomes for each mutation type. Frequency in % is shown in parentheses.

Mutation Type	Count	Median Number of Genomes (%)
in UTRs	1842	120 (2.2 × 10^−3^ %)
non-synonymous	51,467	20 (3.8 × 10^−4^ %)
synonymous	18,413	135 (2.6 × 10^−3^ %)
not alone	1742	1 (1.9 × 10^−5^ %)

**Table 2:** The 10 most common SARS-CoV-2 mutations.

Mutation	Gene	Mutation Type	AA Change	No. Genomes (%)
A23403G	*S*	non-synonymous	D614G	5,312,457 (99.48%)
C14408U	*RdRp*	non-synonymous	P323L	5,306,009 (99.35%)
C3037U	*nsp3*	synonymous	F106F	5,301,673 (99.27%)
C241U	*5’-UTR*	in UTRs	-	5,231,432 (97.96%)
del_28271:1	*N*	deletion	-	3,835,041 (71.81%)
C22995A	*S*	non-synonymous	T478K	3,456,098 (64.71%)
C10029U	*nsp4*	non-synonymous	T492I	3,176,761 (59.48%)
U22917G	*S*	non-synonymous	L452R	3,149,402 (58.97%)
G29402U	*N*	non-synonymous	D377Y	3,105,411 (58.15%)
C23604G	*S*	non-synonymous	P681R	3,097,770 (58.00%)

**Table 3:** Total frequency and variant frequency of some of the *S* mutations used in the classification of SARS-CoV-2 variants.

Mutation	No. Genomes (% ^a^)	No. Genomes (% ^b^) in Variants					
		Alpha	Beta	Delta	Epsilon	Gamma	Omicron
Δ69/70	945,256 (17.70%)	895,448 (94.73%)	14 (0.001%)	3850 (0.41%)	287 (0.03%)	189 (0.02%)	19,260 (2.04%)
W152C	45,304 (0.85%)	9 (0.02%)	0 (0%)	29 (0.06%)	45,192 (99.75%)	2 (0.004%)	1 (0.002%)
K417N	383,722 (7.18%)	137 (0.04%)	24,366 (6.35%)	5353 (1.40%)	10 (0.003%)	2 (0.0005%)	352,543 (91.87%)
K417T	88,480 (16.32%)	32 (0.04%)	0 (0%)	59 (0.07%)	1 (0.001%)	86,902 (98.22%)	1426 (1.61%)
L452R	3,149,260 (58.97%)	450 (0.01%)	28 (0.0009%)	3,075,838 (97.67%)	45,457 (1.44%)	42 (0.001%)	1847 (0.06%)
E484A	357,227 (6.69%)	20 (0.006%)	0 (0%)	2595 (0.73%)	1 (0.0003%)	0 (0%)	354,118 (99.13%)
N501Y	1,396,003 (26.14%)	914,121 (65.48%)	25,377 (1.82%)	1520 (0.11%)	25 (0.0002%)	89,927 (6.44%)	348,897 (24.99%)

^a^ The percentage is calculated in relation to the total number of genomes. ^b^ The percentage of each variant is calculated in relation to the total number of genomes containing that mutation.

**Table 4:** SNV counts showing the initial nucleotide (from) and the new nucleotide (to). The percentage of the total number of initial bases in the SARS-CoV-2 genome is displayed in parentheses.

		To Nucleotide				
		A	G	C	U	Total SNVs
**From nucleotide**	**A**	0	8416 (94.0%)	7008 (78.3%)	6982 (78.0%)	22,406
	**G**	5420 (92.4%)	0	4059 (69.2%)	5475 (93.4%)	14,954
	**C**	4780 (87.0%)	3580 (65.2%)	0	5351 (97.4%)	13,711
	**U**	6791 (70.8%)	6666 (69.5%)	8936 (93.1%)	0	22,393
**Total SNVs**		16,991	18,662	20,003	17,808	73,464

**Table 5:** The number of different mutations found in SARS-CoV-2 regions that hybridize with probes and forward and reverse primers from some COVID-19 diagnostic RT-qPCR tests.

Name	Gene	Region Amplified	No. Different Mutations Found in Forward and Reverse Primers and Probe	Total No. Mutations and Total Frequency (%)
nCoV_IP2	*RdRp*	12,690–12,797	46 | 68 | 64	178 (1.75%)
nCoV_IP4	*RdRp*	14,080–14,186	50 | 66 | 65	181 (3.95%)
Charite-E	*E*	26,269–26,381	89 | 81 | 143	313 (7.15%)
N-Sarbeco	*N*	28,706–28,833	68 | 93 | 116	277 (2.14%)
Charite-RdRp	*RdRp*	15,431–15,528	67 | 52 | 64	183 (60.84%)
HKU-ORF1ab	*ORF1ab*	18,778–18,909	60 | 73 | 60	193 (1.18%)
HKU-N	*N*	29,145–29,254	145 | 222 | 167	534 (3.25%)
China-CDC-ORF1ab	*ORF1ab*	13,342–13,460	58 | 59 | 103	220 (0.79%)
China-CDC-N	*N*	28,881–28,979	156 | 118 | 86	360 (141.28%)
US-CDC-N1	*N*	28,287–28,358	102 | 111 | 131	344 (14.59%)
US-CDC-N2	*N*	29,164–29,230	154 | 184 | 189	527 (2.73%)
US-CDC-N3	*N*	28,681–28,752	88 | 91 | 90	269 (3.36%)
Japan-N	*N*	29,125–29,282	116 | 234 | 211	561 (2.02%)
Thailand-N	*N*	28,320–28,376	104 | 112 | 78	294 (2.46%)
Sigma-Aldrich	*N*	28,750–28,860	96 | 96 | -^1^	192 (2.66%)

^1^ It does not use a probe.

## Data Availability

We have created the database SARS-CoV-2 Mutation Portal (http://sarscov2-mutation-portal.urv.cat/SARS-CoV-2_mutation-portal, accessed on 10 May 2023) with all mutations discovered in the more than five million genomes analyzed.

## Supplementary Materials

The supporting information can be downloaded at: https://www.mdpi.com/article/10.3390/ijms24109072/s1.
