# Supplementary material for: The Mutational Landscape of SARS-CoV-2
Source: Int J Mol Sci. 2023 May 22;24(10):9072. doi: 10.3390/ijms24109072 (PMC10219494; doi:10.3390/ijms24109072)
Supplement: Supplementary file 1 [file ijms-24-09072-s001.zip › ijms-2341634-supplementary.pdf]

Supplementary Information for
The mutational landscape of SARS-CoV-2

Bryan Saldivar-Espinoza[1,†], Pol Garcia-Segura[1], Nil Novau-Ferré[1,†], Guillem Macip[1], Ruben Martinez[2], Pere Puigbò [3,4,5], Adrià Cereto-Massagué[6], Gerard Pujadas[1,*] and Santiago Garcia-Vallve[1,*]

[1] Departament de Bioquímica i Biotecnologia, Research group in Cheminformatics & Nutrition, Campus de Sescelades, Universitat Rovira i Virgili, 43007 Tarragona, Spain; bsaldivar.emc2@gmail.com (B.S.-E.); polgarse2@gmail.com (P.G.-S.); nnovauf@gmail.com (N.N.-F.); guillem.macip@gmail.com (G.M.)

[2] Institut La Guineueta. 08042 Barcelona, Spain; rmartbernabe@gmail.com

[3] Department of Biology, University of Turku, 20500 Turku, Finland; pepuav@utu.fi

[4] Department of Biochemistry and Biotechnology, Rovira i Virgili University, 43007 Tarragona, Spain

[5] Eurecat Technology Centre of Catalonia, Unit of Nutrition and Health, 43204 Reus, Spain

[6] EURECAT Centre Tecnològic de Catalunya, Centre for Omic Sciences (COS), Joint Unit Universitat Rovira i Virgili-EURECAT, Unique Scientific and Technical Infrastructures (ICTS), 43204 Reus, Spain. ssorgatem@gmail.com

This PDF file includes:

Figures S1 to S14
Tables S1 to S3

Figure S1. Bar chart of the percentage of analyzed genomes sequenced for each country.

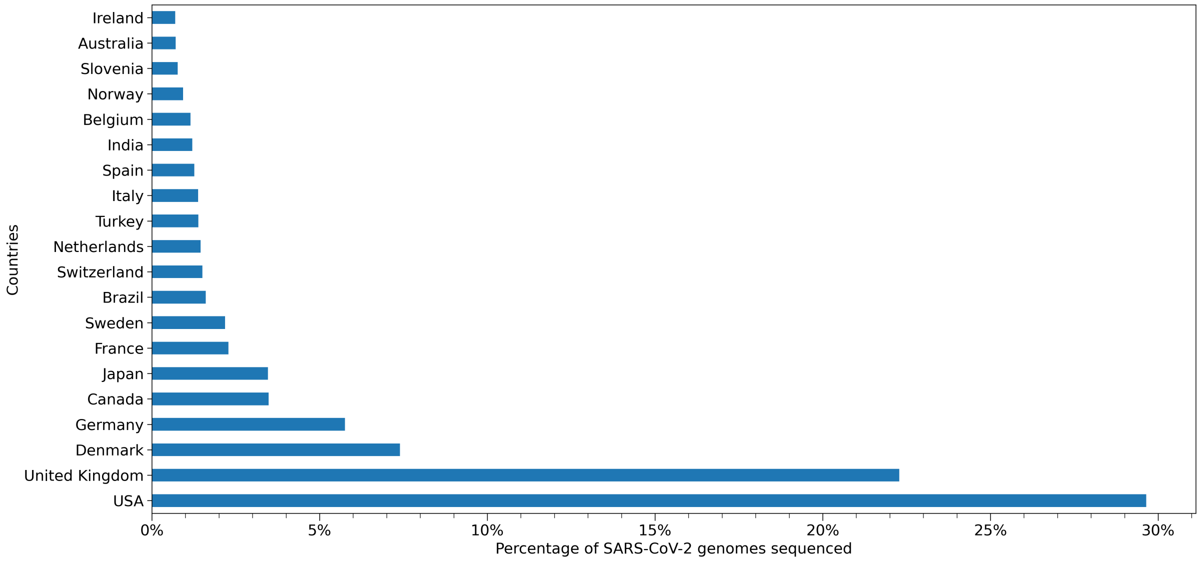

Figure S2. Mean number of SNVs per genome and week.

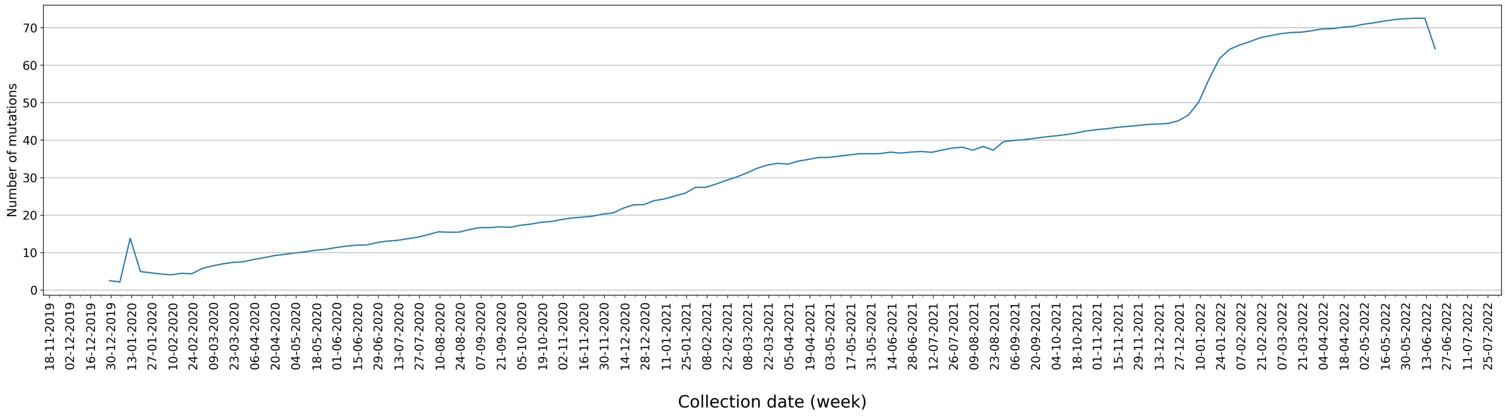

Figure S3. Mean number of deletions per genome and week.

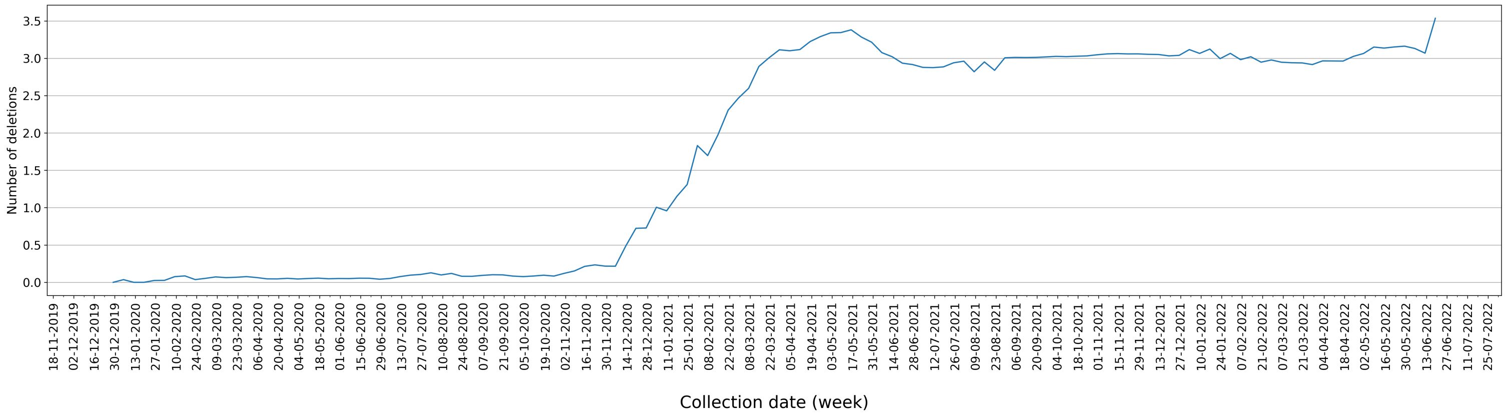

Figure S4. Mean number of insertions per genome and week.

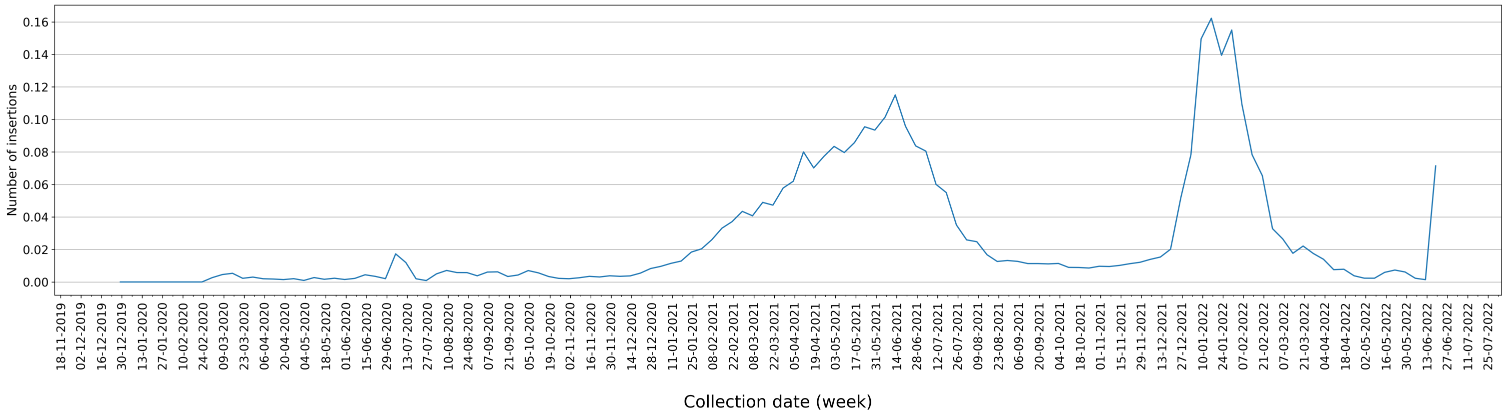

Figure S5. Histogram of the length of deletions in SARS-CoV-2 genomes. Only deletions with a relative frequency greater than 10% were analyzed.

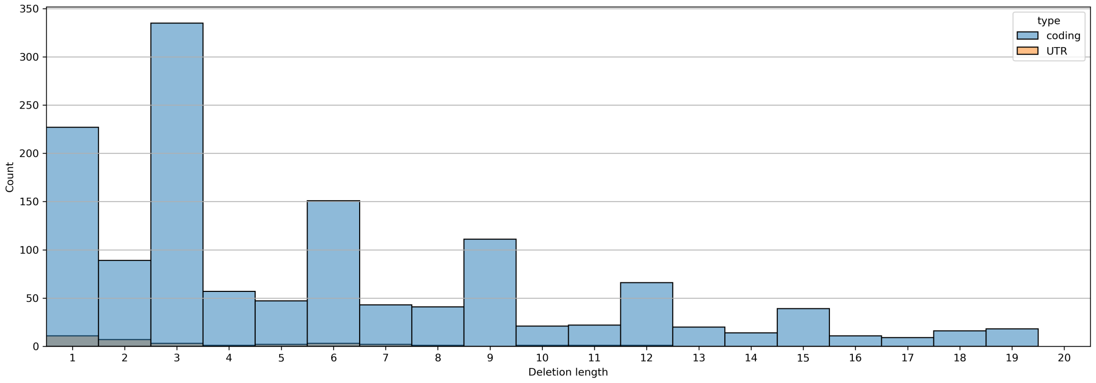

Figure S6. Histogram of the length of insertions in SARS-CoV-2 genomes. Only deletions with a relative frequency greater than 10% were analyzed.

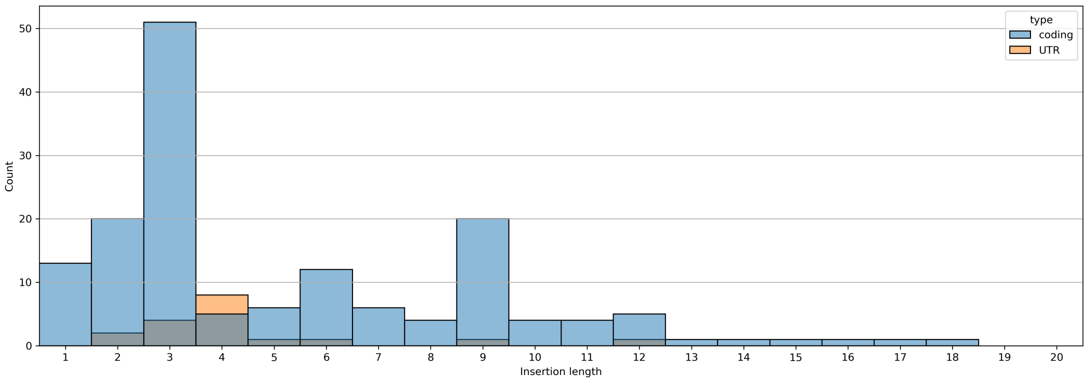

Figure S7. Box plots of the relative frequency of SNV types.

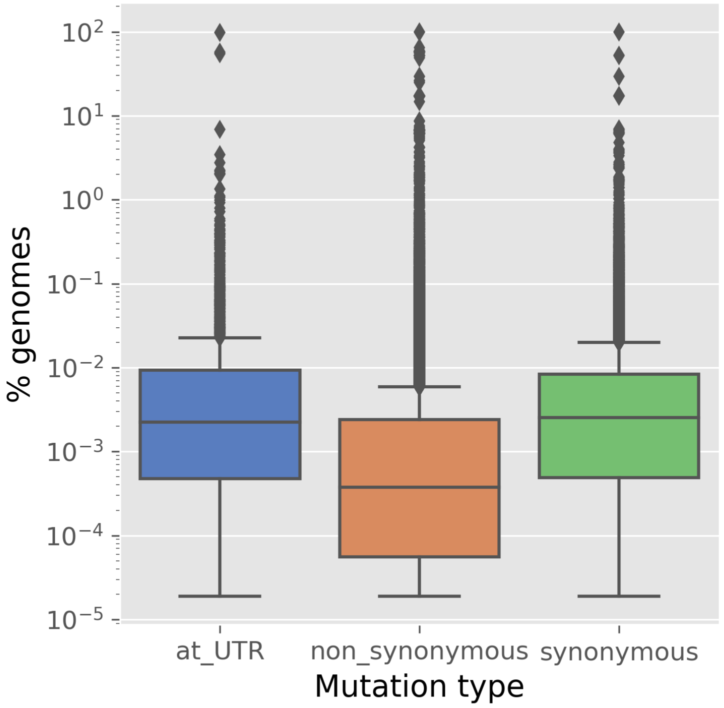

Figure S8. Histogram of the percentage of SNVs found in a given number of genomes.

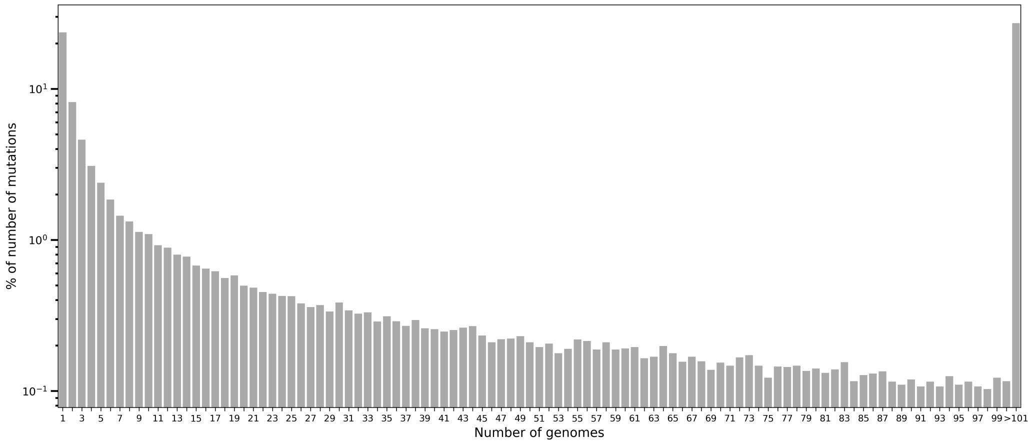

Figure S9. Lolliplots of the most frequent mutations in each SARS-CoV-2 gene. Synonymous, non-synonymous and UTR mutations are shown in green, dark orange and blue, respectively. Deletions are shown in purple.

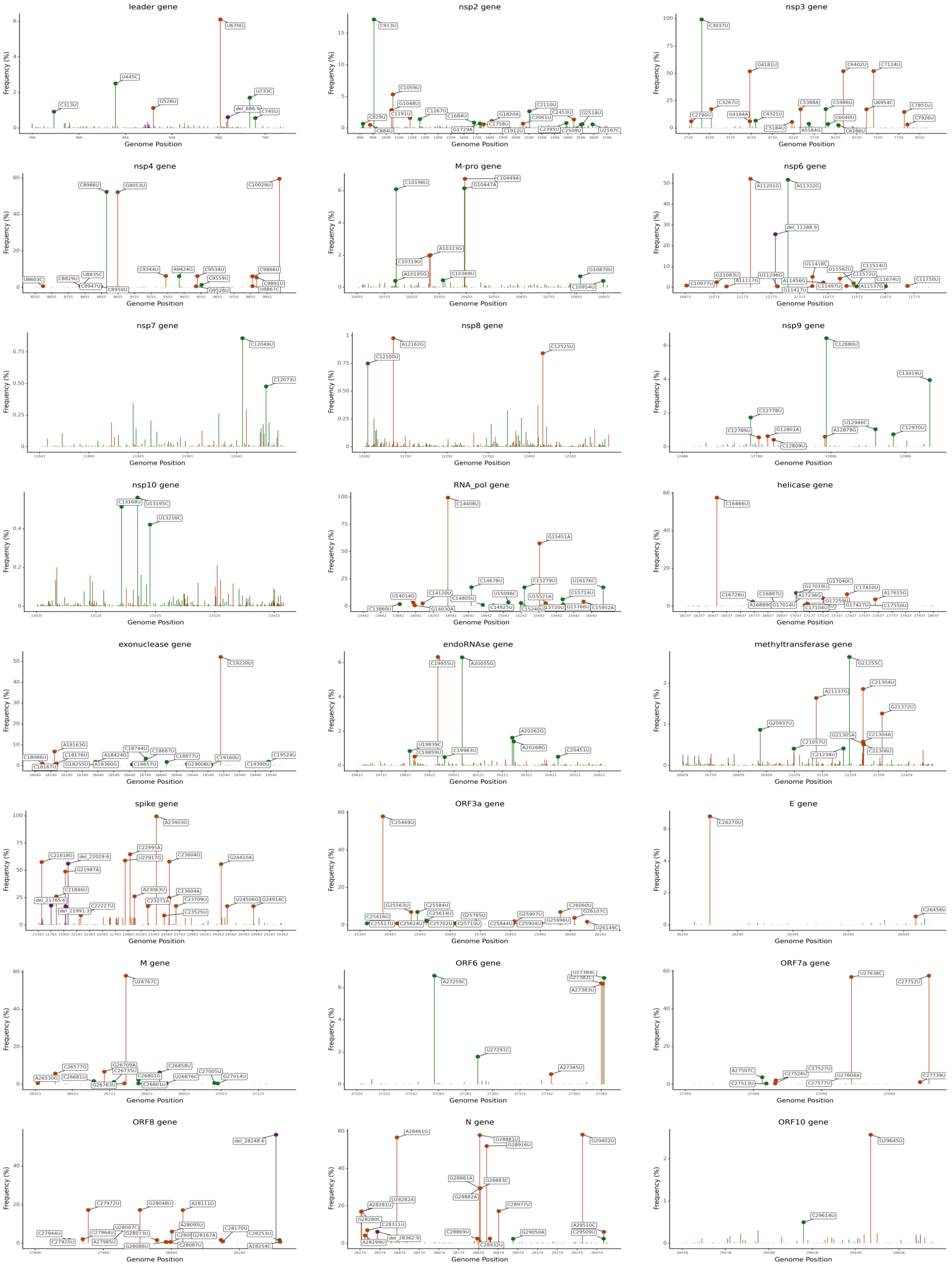

Figure S10. Box plots of the frequency (A), number of pangolin lineages (B), number of countries (C) and number of variants (D) of SNV types.

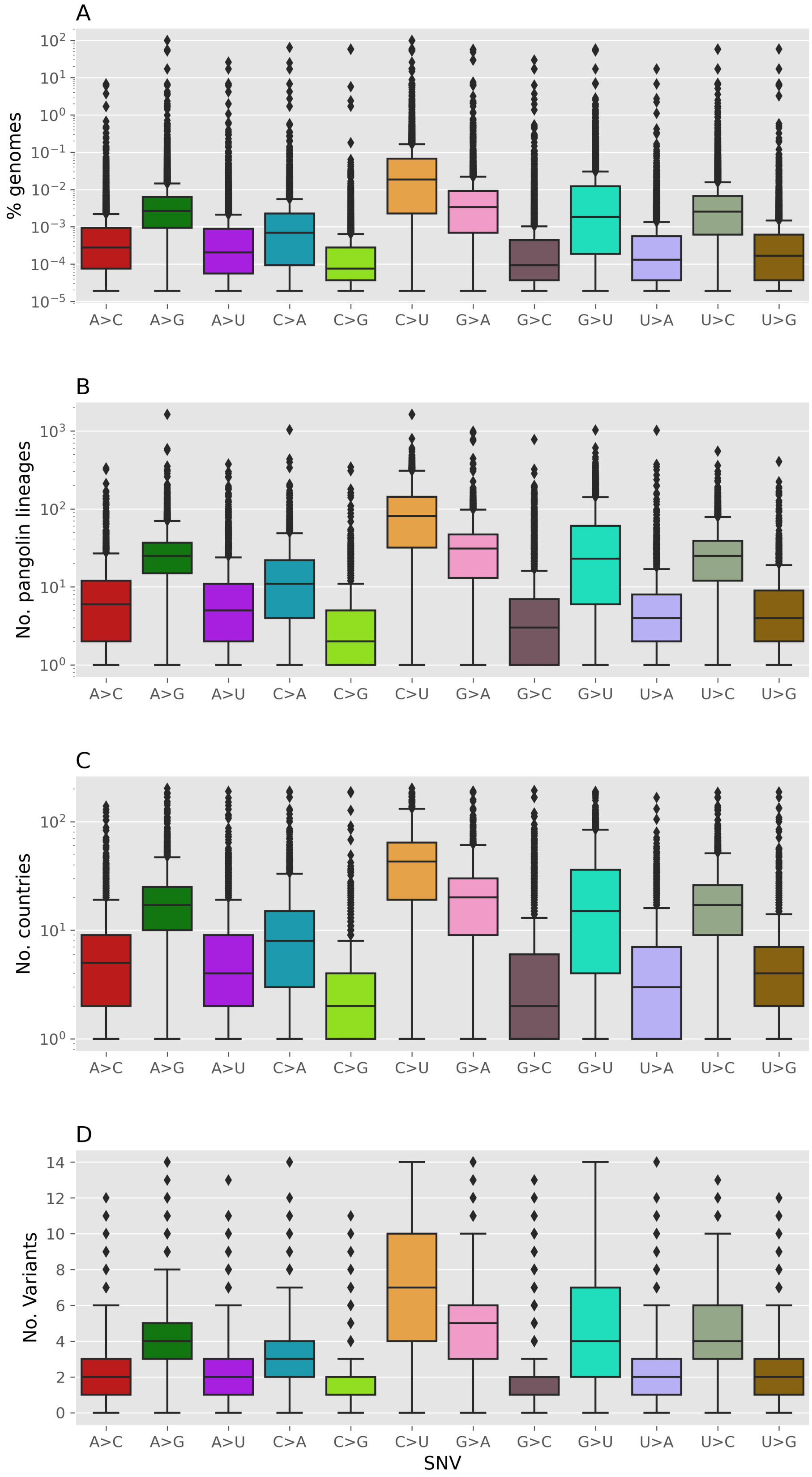

Figure S11. Box plots of the frequency (%) of the mutations found in SARS-CoV-2 regions that hybridize with probes and forward and reverse primers from some COVID-19 diagnostic PCR tests.

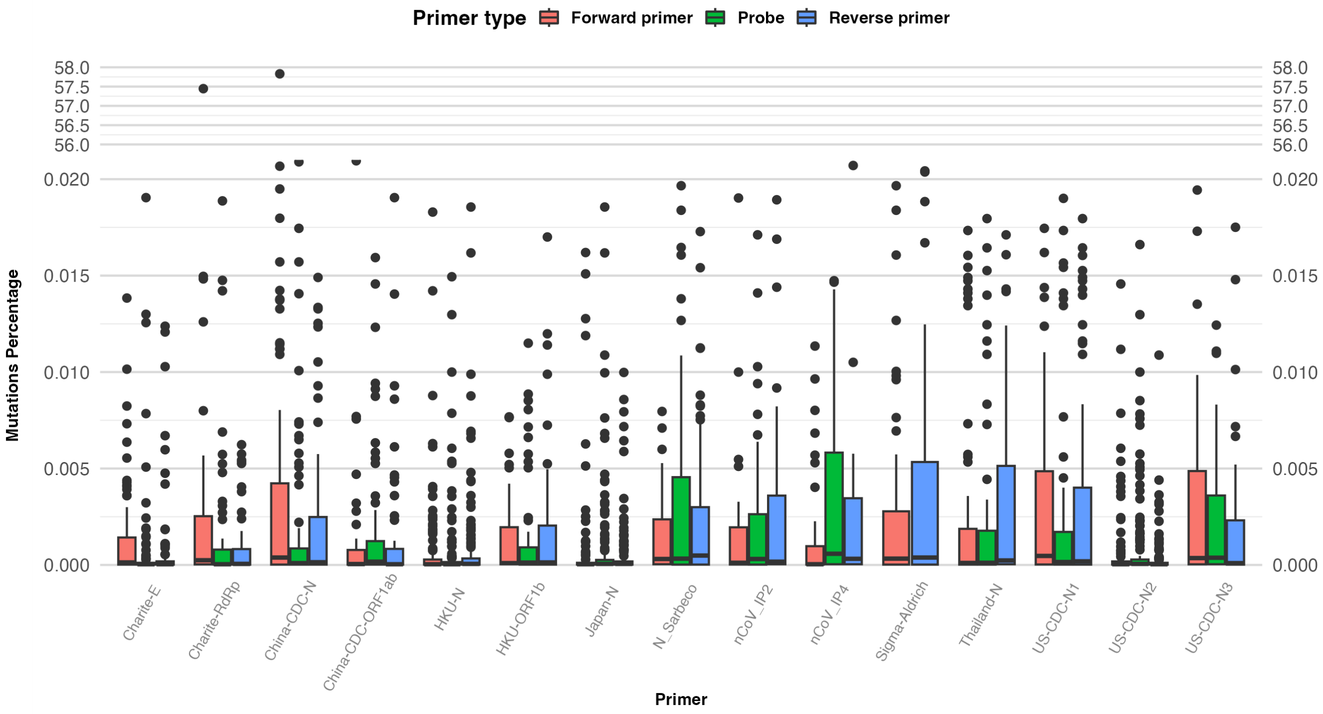

Figure S12. Search engine of the SARS-CoV-2 mutation portal at http://sarscov2-mutation-portal.urv.cat/

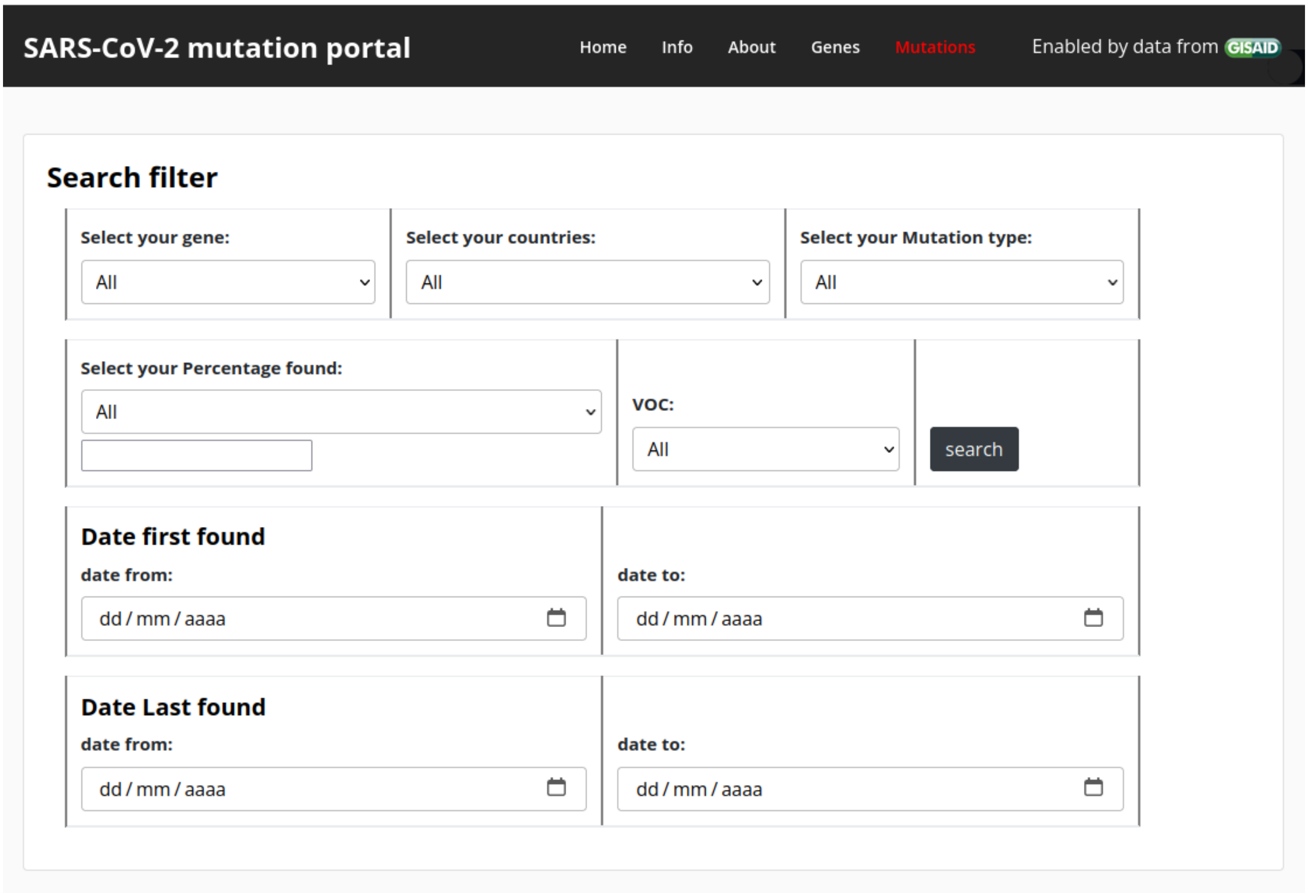

Figure S13. Results of a search of the SARS-CoV-2 mutation portal at http://sarscov2-mutation-portal.urv.cat/

SARS-CoV-2 mutation portal   Home Info About Genes Mutations   Enabled by data from GISAID

Show 10 entries   Search:

mutation	position	gene	muts type	codon	aa	codon position	num found	percentage found	n countries	date first found	id first found
G204T	204	5_UTR	at_UTR				72122	1.35046	98	2020-03-02	EPI_ISL_2013149
G210T	210	5_UTR	at_UTR				3063257	57.35825	187	2020-03-03	EPI_ISL_2758215
C241T	241	5_UTR	at_UTR				5231432	97.95645	202	2020-01-01	EPI_ISL_4405694
T445C	445	leader	synonymous	GTT60GTC	V60V	3	133531	2.50031	92	2020-02-14	EPI_ISL_1014733
G526T	526	leader	missense	GAG87GAT	E87D	3	60108	1.1255	103	2020-03-02	EPI_ISL_10431154
T670G	670	leader	missense	AGT135AGG	S135R	3	325823	6.1009	109	2020-03-28	EPI_ISL_12269393
T733C	733	leader	synonymous	GAT156GAC	D156D	3	91013	1.70418	86	2020-04-23	EPI_ISL_2612488
C913T	913	nsp2	synonymous	TCC36TCT	S36S	3	915334	17.13926	169	2020-03-12	EPI_ISL_2898105
G1048T	1048	nsp2	missense	AAG81AAT	K81N	3	151117	2.82961	140	2020-05-11	EPI_ISL_6463150
C1059T	1059	nsp2	missense	ACC85ATC	T85I	2	282636	5.29225	160	2020-01-01	EPI_ISL_4405694

Showing 1 to 10 of 247 entries   Previous 1 2 3 4 5 …

Figure S14. Scatter plot of the above search

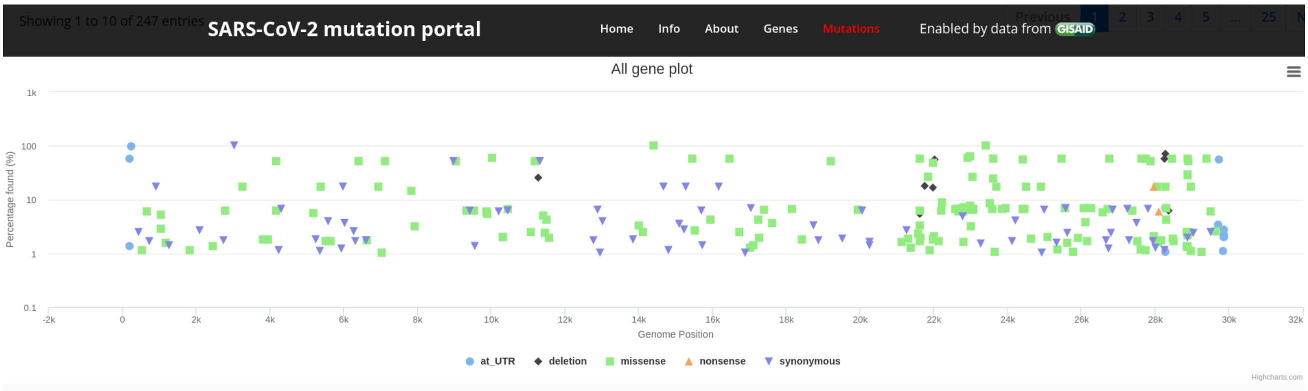

Table S1. Distribution by continent of the 5,340,569 SARS-CoV-2 genomes analyzed.

Continent	Genomes sequenced	Percentage (%)
Europe	2,942,990	55.1
North America	1,819,981	34.1
Asia	371,704	7.0
South America	126,872	2.4
Oceania	44,942	0.8
Africa	34,080	0.6

Table S2. Number of different SNVs, insertions and deletions found in the 5,340,569 SARS-CoV-2 genomes analyzed.

Mutation	Total number[1]	Coding regions	UTRs
SNVs	73,464	71,622	1842
deletions	21,712	21,464	248
insertions	1820	1700	120

[1]Number of different SNV, deletions or insertions

Table S3. Mutations found in SARS-CoV-2 regions that hybridize with probes and forward and reverse primers from some COVID-19 diagnostic PCR tests.

Name	Gene	Forward primer position	Forward primer count[2]	Forward primer %[1]	Forward primer 5 last count[2,3]	Forward primer 5 last %[1,3]	Reverse primer position	Reverse primer count[2]	Reverse primer %[1]	Reverse primer 5 last count[2,3]	Reverse primer 5 last %[1,3]	Probe position	Probe count[2]	Probe %[1]	Total count	Total %[1]
nCoV_IP2	RdRp	12,690-12,707	46 (5|0)	0.07	14 (1|0)	0.039	12,780-12,797	68 (13|2)	1.25	18 (5|1)	0.14	12,717-12,737	64 (12|0)	0.44	178	1.75
nCoV_IP4	RdRp	14,080-14,098	50 (10|0)	0.12	13 (3|0)	0.068	14,167-14,186	66 (6|0)	0.85	15 (2|0)	0.038	14,105-14,123	65 (4|1)	2.97	181	3.94
Charite-E	E	26,269-26,294	89 (16|0)	6.91	15 (5|0)	0.018	26,360-26,381	81 (22|1)	0.10	30 (15|1)	0.003	26,332-26,357	143 (32|0)	0.14	313	7.15
N_Sarbeco	N	28,706-28,724	68 (8|0)	0.87	22 (5|0)	0.23	28,814-28,833	93 (18|0)	0.75	34 (14|0)	0.17	28,753-28,777	116 (23|3)	0.52	277	2.14
Charite-RdRp	RdRp	15,431-15,452	67 (10|0)	57.85	24 (5|0)	57.57	15,505-15,528	52 (9|0)	2.76	15 (6|0)	0.01	15,470-15,494	64 (15|0)	0.23	183	60.84
HKU-ORF1b	ORF1ab	18,778-18,797	60 (3|0)	0.31	11 (0|0)	0.07	18,889-18,909	73 (13|0)	0.70	19 (3|0)	0.14	18,849-18,872	60 (5|0)	0.17	193	1.18
HKU-N	N	29,145-29,166	145 (79|0)	0.59	93 (77|0)	0.08	29,236-29,254	222 (151|0)	1.95	154 (136|0)	0.16	29,177-29,196	167 (104|1)	0.71	534	3.25
China-CDC-ORF1ab	ORF1ab	13,342-13,362	58 (11|1)	0.27	12 (4|0)	0.009	13,442-13,460	59 (13|0)	0.24	16 (6|0)	0.04	13,377-13,404	103 (21|1)	0.29	220	0.79
China-CDC-N	N	28,881-28,902	156 (35|3)	120.51	48 (23|2)	0.26	28,958-28,979	118 (23|4)	20.42	30 (13|0)	0.56	28,934-28,953	86 (20|2)	0.35	360	141.28
US-CDC-N1	N	28,287-28,306	102 (15|4)	5.29	26 (7|0)	0.06	28,335-28,358	111 (23|2)	0.56	29 (10|1)	0.11	28,309-28,332	131 (25|2)	8.75	344	14.59
US-CDC-N2	N	29,164-29,183	154 (92|1)	1.11	104 (90|1)	0.50	29,213-29,230	184 (131|0)	0.66	129 (117|0)	0.13	29,188-29,210	189 (115|0)	0.95	527	2.73
US-CDC-N3	N	28,681-28,702	88 (14|0)	0.96	22 (7|0)	0.11	28,732-28,752	91 (17|2)	0.75	25 (8|0)	0.15	28,704-28,727	90 (10|0)	1.64	269	3.36
Japan-N	N	29,125-29,144	116 (63|0)	0.52	77 (60|0)	0.35	29,263-29,282	234 (173|0)	0.92	174 (159|0)	0.24	29,222-29,241	211 (140|0)	0.59	561	2.02
Thailand-N	N	28,320-28,339	104 (21|2)	1.25	27 (8|1)	0.09	28,358-28,376	112 (29|0)	0.82	35 (17|0)	0.17	28,341-28,356	78 (18|1)	0.39	294	2.46
Sigma-Aldrich	N	28,750-28,771	96 (18|1)	0.39	26 (11|0)	0.10	28,842-28,860	96 (17|2)	2.27	30 (8|0)	0.14	-	0	0	192	2.66

[1] Total accumulated frequencies in %. [2] The numbers in brackets indicate the number of deletions and insertions. [3]Only the last 5 bases of the 3′-end have been taken into account
